# Pregnancy outcomes of intrauterine insemination without ovarian stimulation in couples affected by unilateral tubal occlusion and male infertility

**DOI:** 10.1186/s12884-023-05705-3

**Published:** 2023-05-24

**Authors:** Yan Tang, Yu-Xia He, Yun Ye, Ting-Ting Zhang, Jing-Jing Wang, Qian-Dong He

**Affiliations:** 1 Center for Reproductive Medicine, Department of Gynecology and Obstetrics, Zhongshan City People’s Hospital, Zhongshan, 528403 Guangdong Province China; 2 Department of Obstetrics and Gynecology, Center for Reproductive Medicine, Guangdong Provincial Key Laboratory of Major Obstetric Diseases, The Third Affiliated Hospital of Guangzhou Medical University, Guangzhou, 510150 Guangdong Province China

**Keywords:** Unilateral tubal occlusion, Ovarian stimulation, Intrauterine insemination, Clinical pregnancy, Live birth, First trimester miscarriage

## Abstract

**Background:**

Information available to date regarding the pregnancy outcomes of intrauterine insemination (IUI) without ovarian stimulation (OS) in infertile patients with unilateral tubal occlusion remains scarce. The objectives of this study were to investigate for couples affected by unilateral tubal occlusion (diagnosed via hysterosalpingography (HSG)/transvaginal real-time three-dimensional hysterosalpingo-contrast sonography (TVS RT-3D-HyCoSy)) and male infertility: (1) whether significant differences exist in pregnancy outcomes between IUI with or without OS cycles, and (2) whether the pregnancy outcomes of IUI without OS in women with unilateral tubal occlusion were similar to those of women with bilateral patent tubes.

**Methods:**

258 couples affected by male infertility completed 399 IUI cycles. The cycles were divided into three groups: group A, IUI without OS in women with unilateral tubal occlusion; group B, IUI with OS in women with unilateral tubal occlusion; and group C, IUI without OS in women with bilateral patent tubes. The main outcome measures, including clinical pregnancy rate (CPR), live birth rate (LBR), and first trimester miscarriage rate, were compared between either groups A and B or groups A and C.

**Results:**

Although the number of dominant follicles > 16 mm were significantly higher in group B than that in group A (group B vs. group A: 1.6 ± 0.6 vs. 1.0 ± 0.2, *P* < 0.001), the CPR, LBR, and first trimester miscarriage rate were comparable between these two groups. When comparing group C to group A, the duration of infertility was significantly longer in group C than that in group A (group A vs. group C: 2.3 ± 1.2 (year) vs. 2.9 ± 2.1 (year), *P* = 0.017). Except for the first trimester miscarriage rate, which was significantly higher in group A (42.9%, 3/7) than that in group C (7.1%, 2/28) (*P* = 0.044), no significant differences were observed in the CPR and LBR in these two groups. After adjusting for female age, body mass index, and the duration of infertility, similar results were obtained between groups A and C.

**Conclusions:**

In couples affected by unilateral tubal occlusion (diagnosed via HSG/TVS RT-3D-HyCoSy) and male infertility, IUI without OS might be an alternative treatment strategy. However, when compared to patients with bilateral patent tubes, the patients with unilateral tubal occlusion showed a higher first trimester miscarriage rate following IUI without OS cycles. Further studies are warranted to clarify this relationship.

**Supplementary Information:**

The online version contains supplementary material available at 10.1186/s12884-023-05705-3.

## Background

Intrauterine insemination (IUI) is an effective and frequently used fertility treatment worldwide in the management of couples with unexplained and mild male factor infertility. It is less invasive, less stressful, more cost-effective, and more easily accepted by patients than other available treatments [1–3]. IUI can be performed with or without ovarian stimulation (OS).

For IUI, at least one patent fallopian tube is required. There are multiple approaches to diagnose tubal patency, including hysterosalpingography (HSG), hysterosalpingo-contrast sonography (HyCoSy), and laparoscopy with dye chromopertubation. Although laparoscopy with dye chromopertubation remains the gold-standard test in the diagnosis of tubal pathologies, HSG and HyCoSy are widely used as the first-line approach to assess the anatomy of the uterus and the patency of the fallopian tubes in women with infertility [4–7].

Compared to bilateral patent tubes or bilateral tubal occlusion, the diagnosis of unilateral tubal pathology has more limited prognostic significance and management strategies. One management strategy is IUI with OS through the available patent tube. Several studies [8–12] have demonstrated that the clinical pregnancy rate (CPR) and/or cumulative pregnancy rate after IUI with OS in patients diagnosed with unilateral tubal occlusion using HSG were similar to those with bilateral tubal patency and unexplained infertility. Therefore, IUI with OS is reccomended as the initial treatment in infertile women with unilateral tubal occlusion. However, could IUI without OS be an alternative treatment for infertile patients with unilateral tubal occlusion? Information available to date regarding the pregnancy outcomes of IUI without OS in infertile patients with unilateral tubal occlusion remains scarce. Therefore, the aims of the present study were to investigate for couples affected by unilateral tubal occlusion and male infertility: (1) whether significant differences exist in pregnancy outcomes between IUI with or without OS cycles, and (2) whether the pregnancy outcomes of IUI without OS in women with unilateral tubal occlusion were similar to those of women with bilateral patent tubes.

## Methods

### Patients

This retrospective study reviewed the clinical data of 838 couples who underwent IUI treatment for infertility at the Center for Reproductive Medicine of Zhongshan City People’s Hospital between January 2014 and October 2021. Patients with fallopian tubal patency assessed by HSG or transvaginal real-time three-dimensional hysterosalpingo-contrast sonography (TVS RT-3D-HyCoSy) were investigated. The inclusion criteria were as follows: (1) couples affected by male infertility, which was defined as one or more subnormal semen variables: total sperm < 39 × 10^6^/ejaculation or sperm concentration < 15 × 10^6^/mL; progressive motility < 32%; and normal morphology < 4%, according to World Health Organization criteria (5th version); (2) female patients aged 20–40 years with a normal fertility status; (3) female patients with unilateral tubal occlusion; and (4) females with bilateral patent tubes (only those undergoing IUI without OS cycles were included). Patients were excluded if tubal patency was assessed by laparoscopy with dye chromopertubation. In addition, patients with a history of previous surgery (except for caesarean section) were excluded.

The study protocol was approved by the institutional ethics committee of Zhongshan City People’s Hospital, and informed consent was obtained from all participants for their clinical data.

#### TVS RT-3D-HyCoSy procedure

The TVS RT-3D-HyCoSy procedure was performed by an experienced sonographer. All ultrasound examinations were performed using the Voluson E8 Expert (GE Medical Systems) ultrasound machine with dedicated 3D imaging software and coded contrast imaging (CCI) mode. The detailed process of TVS RT-3D-HyCoSy, as described in the report by Zhou et al. [13], was carried out as follows: A 2D transvaginal ultrasound examination of the uterus, endometrium, adnexa and pouch of Douglas were initially performed. Then a Foley catheter was fixed in the uterine cavity by injecting 2–3 mL normal saline into the balloon of the Foley catheter. The transvaginal volume probe was reintroduced into the vagina and positioned in such a way as to visualize the transverse section of the uterus and, if possible, both ovaries laterally. Switched on the CCI mode, and placed the 3D volume box over the pelvic region. The region of 3D volume acquisition was made as wide as possible, so that the uterus, both ovaries and the whole length of the fallopian tube could be visualized. A 20-mL syringe fully loaded with the diluted contrast medium (SonoVue) was injected into the uterine through the Foley catheter. The flow of the contrast medium in uterus and fallopian tubes, as well as the diffusion around ovary and pelvic cavity were dynamically observed and recorded, and the angiographic data was stored in the instrument hard disk for subsequent analysis. Subsequently, a view of the uterine cavity in the coronal section with both tubes laterally and a view of any rings of spilled contrast medium (if the tube was patent) around the ovaries were observed in 2D mode. Rotating the volume image permitted better visualization of the tubal course in 3D space.

#### Diagnosis criteria for patent fallopian tubes

For TVS RT-3D-HyCoSy, a direct sign of tubal patency [13, 14] is visualization of steady cord-like echogenic signals of the contrast agent within the fallopian tube lumen from the interstitial to the infundibular portion, with a rim of contrast material partially or completely surrounding the ipsilateral ovary (ovarian rim sign), which represents free intraperitoneal spill. No obvious resistance exist, or resistance disappeared while maintaining constant pressure on the syringe for 1 min during injection of the contrast medium. While for HSG, opacification of all fallopian tube segments, with subsequent free intraperitoneal spill, is a sign of tubal patency [14].

### Ovarian stimulation protocols, follicle monitoring, and follow-up

In our center, mild OS (1–2 follicles) were recommended. The OS protocols, as documented in our previous study [15], were as follows: (1) clomiphene citrate (CC) or letrozole (LE) for 5 days; (2) gonadotrophins (Gn) for a variable duration depending on patient response; and (3) CC/LE combined with Gn for a variable duration depending on patient response. All protocols began on days 3–5 of the menstrual cycle. Follicular development was monitored using transvaginal ultrasonography on days 8–10, and this was repeated every 2 or 3 days based on the follicular size. Couples were advised to cancel the cycle if more than three dominant follicles > 16 mm were present or if all dominant follicles appeared on the ipsilateral ovary of the blocked fallopian tube. Ovulation was triggered when at least one follicle ≥ 18 mm was seen on transvaginal ultrasonography. If an endogenous luteinizing hormone surge occurred (> 20 IU), the trigger was omitted. IUI was performed within 24–48 h, according to the serum luteinizing hormone and progesterone levels.

Biochemical pregnancy was confirmed by measuring serum β-human chorionic gonadotropin levels on days 14–16 after IUI. In women with positive β-human chorionic gonadotropin levels, transvaginal ultrasound examination was performed 2 weeks later to confirm clinical pregnancy. The outcomes of clinical pregnancy were subsequently recorded, including spontaneous abortion, ectopic pregnancy, multiple pregnancies, and live birth. The main outcome measures were CPR, live birth rate (LBR), and first trimester miscarriage rate. Clinical pregnancy was defined as the observation of a gestational sac inside or outside the uterine cavity using transvaginal ultrasonography. Live birth was defined as the birth of an infant after at least 28 weeks of gestation, with postnatal evidence of life. First trimester miscarriage was defined as spontaneous pregnancy loss within the first 12 weeks of gestation.

### Statistical analysis

Statistical analysis was performed using the Statistical Package for Social Science (SPSS, version 25.0; IBM, Armonk, NY, USA). Continuous variables were expressed as mean ± standard deviation (SD). The normality of data was evaluated. Independent-Samples T-test was used for the comparison of normally distributed data, while Mann-Whitney U-test was used for non-normally distributed data. Categorical data, described as frequencies and percentages, were compared by the chi-square test or Fisher’s exact test, as appropriate. A binary logistic regression model was used to adjust for potential confounders to explore whether unilateral tubal occlusion was related to the pregnancy outcomes (clinical pregnancy/live birth/first trimester miscarriage) of IUI without OS. Statistical significance was set at *P* < 0.05.

## Results

A total of 258 couples were recruited for this study. Among them, 80 women had unilateral tubal occlusion, and the remainder were diagnosed with bilateral patent tubes. Together they completed 399 IUI cycles. The CPR/cycle was 10.3% (41/399), the LBR/cycle was 8.3% (33/399), and the first trimester miscarriage rate was 12.2% (5/41). No ectopic pregnancies or multiple pregnancies occurred. The cycles were divided into three groups: group A (n = 75), IUI without OS in patients with unilateral tubal occlusion; group B (n = 66), IUI with OS in patients with unilateral tubal occlusion; and group C (n = 258), IUI without OS in patients with bilateral patent tubes.

The basic clinical parameters of groups A and B were compared (Table 1). Besides the number of dominant follicles > 16 mm, which were significantly higher in group B (group B vs. group A: 1.6 ± 0.6 vs. 1.0 ± 0.2, *P* < 0.001), no significant differences were found. The CPR, LBR, and first trimester miscarriage rate were also comparable between the two groups.

**Table 1:** Basic clinical parameters and pregnancy outcomes of three study groups

	Group A	Group B	Group C	*P*-value (A vs. B)	*P*-value (A vs. C)
N	75	66	258		
Female age (year)	30.4 ± 4.3	30.2 ± 4.8	31.1 ± 4.0	0.585	0.310
Male age (year)	33.2 ± 6.1	32.8 ± 5.9	33.3 ± 5.3	0.679	0.895
Body mass index (BMI, kg/m2)	21.0 ± 2.6	21.1 ± 2.7	20.9 ± 2.7	0.478	0.910
Type of infertility (n (%))				0.851	0.834
Primary	42 (56.0)	38 (57.6)	148 (57.4)		
Secondary	33 (44.0)	28 (42.4)	110 (42.6)		
Duration of infertility (year)	2.3 ± 1.2	2.4 ± 1.1	2.9 ± 2.1	0.298	0.017^*^
Basal FSH (mIU/ml)	6.5 ± 1.9	6.4 ± 2.2	6.5 ± 2.0	0.919	0.903
No. of dominant follicles > 16 mm	1.0 ± 0.2	1.6 ± 0.6	1.0 ± 0.1	0.000^*^	0.186
Endometrium thickness (mm)	10.1 ± 1.7	10.0 ± 1.9	10.1 ± 1.6	0.724	0.969
Total progressive motile sperm count (post-wash)	19.3 ± 11.4	22.4 ± 13.1	18.3 ± 11.2	0.243	0.512
Normal morphology (%, post-wash)	4.7 ± 1.9	4.8 ± 1.6	4.4 ± 1.9	0.394	0.160
tubal occlusion site				0.497	/
proximal tubal occlusion	53 (70.7)	50 (75.8)	/		
mid-distal or distal tubal occlusion	22 (29.3)	16 (24.2)	/		
Clinical pregnancy (n (%))	7 (9.3)	6 (9.1)	28 (10.9)	0.960	0.706
Live birth (n (%))	4 (5.3)	4 (6.1)	25 (9.7)	1.000	0.239
First trimester miscarriage (n (%))	3 (42.9)	0 (0)	2 (7.1)	0.192	0.044^*^

Group A: IUI without ovarian stimulation (OS) in patients with unilateral tubal occlusion; Group B: IUI with OS in patients with unilateral tubal occlusion; and Group C: IUI without OS cycle in patients with bilateral patent tubes. Values are presented as mean ± standard deviation or n (%)

*P < 0.05 was considered statistically significant

Basic clinical parameters were comparable between groups A and C except for the duration of infertility, which was significantly longer in group C (group A vs. group C: 2.3 ± 1.2 (year) vs. 2.9 ± 2.1 (year), *P* = 0.017) (Table 1). No significant differences were observed in the CPR and LBR. However, the first trimester miscarriage rate was significantly higher in group A (42.9%, 3/7) than that in group C (7.1%, 2/28) (*P* = 0.044). In the binary logistic regression model, only body mass index (BMI) was found to be significantly related to clinical pregnancy (odds ratio [OR] 1.131, 95% confidence interval [CI]: 1.005–1.273, *P* = 0.041) and live birth (OR 1.139, 95% CI: 1.005–1.290, *P* = 0.042), while female age (OR 1.981, 95% CI: 1.064–3.687, *P* = 0.031) was shown to be significantly related to miscarriage in the first trimester. After adjusting for female age, BMI, and the duration of infertility, no significant differences were observed in CPR or LBR between groups A and C, except for the first trimester miscarriage rate (OR 0.013, 95% CI: 0.000–0.891, *P* = 0.044) (Table 2).

**Table 2:** Binary logistic regression analysis of factors related to pregnancy outcomes in patients underwent IUI without ovarian stimulation

Variables	β	SE (β)	Wald χ2	*P-value*	OR (95%CI)
*Clinical pregnancy*					
Female age	0.036	0.044	0.683	0.408	1.037 (0.952–1.129)
Body mass index (BMI)	0.123	0.060	4.179	0.041 ^*^	1.131 (1.005–1.273)
Duration of infertility	-0.121	0.106	1.309	0.253	0.886 (0.720–1.090)
Unilateral/bilateral patent tube	0.212	0.452	0.220	0.639	1.236 (0.510–2.997)
*Live birth*					
Female age	-0.020	0.048	0.168	0.682	0.980 (0.892–1.077)
Body mass index (BMI)	0.130	0.064	4.132	0.042 ^*^	1.139 (1.005–1.290)
Duration of infertility	-0.099	0.113	0.775	0.379	0.905 (0.726–1.130)
Unilateral/bilateral patent tube	0.732	0.563	1.689	0.194	2.079 (0.690–6.267)
*First trimester miscarriage*					
Female age	0.683	0.317	4.648	0.031 ^*^	1.981 (1.064–3.687)
Body mass index (BMI)	0.000	0.367	0.000	0.999	1.000 (0.487–2.053)
Duration of infertility	-1.926	1.403	1.884	0.170	0.146 (0.009–2.280)
Unilateral/bilateral patent tube	-4.337	2.154	4.054	0.044 ^*^	0.013 (0.000–0.891)

* *P* < 0.05 was considered statistically significant

To eliminate the potential influence of repeated cycles on the results, the same analyses were performed including only the first cycle. Similar results were observed between groups A and B and between groups A and C (Supplemental Table).

## Discussion

To the best of our knowledge, this is the first study to investigate the pregnancy outcomes of IUI without OS in couples affected by unilateral tubal occlusion (diagnosed by HSG or TVS RT-3D-HyCoSy) and male infertility. The results showed that in patients with unilateral tubal occlusion diagnosed by HSG or TVS RT-3D-HyCoSy, although the number of dominant follicles > 16 mm was significantly higher in IUI with OS cycles, the pregnancy outcomes of IUI without OS cycles, including CPR, LBR, and first trimester miscarriage rate, were comparable to those of IUI with OS cycles.

Assessing tubal patency is fundamental to evaluate female fertility, as it determines the treatment course. The management of patients with normal bilateral patent tubes or bilateral tubal occlusion diagnosed by HSG/HyCoSy is clear and straightforward. However, this is different when it comes to women with only one confirmed patent tube. Three possible approaches have been suggested [9]: (1) further surgery to achieve recanalization including laparoscopy, catheterization, and operative hystero-falloposcopy; (2) proceeding directly to in vitro fertilization to bypass the problem; and (3) IUI with OS using the available patent tube. Several studies [8–12] have indicated that IUI with OS should be suggested as the initial treatment of choice in patients with unilateral tubal occlusion. The rationale for using OS may be to correct subtle ovulatory dysfunctions not detectable by standard infertility evaluations and to increase the number of oocytes available for fertilization.

Recently, the results from a network meta-analysis [16] showed that the relative risk (RR) for live birth/ongoing pregnancy rates comparing IUI with CC to natural cycle IUI was 1.05 (95% CI 0.63–1.77), while comparing IUI with LE to natural cycle IUI was 1.15 (95% CI 0.63–2.08) and comparing IUI with Gn to natural cycle IUI was 1.46 (95% CI 0.92–2.30). But women undergoing OS protocol were also at risk for multiple pregnancies. It has been reported that multifollicular growth results in significantly higher pregnancy rates than monofollicular growth (15% vs. 8.4%), at the same time, multiple pregnancy rates increased from 3.7 to 17.0% per cycle [17]. Compared to singleton pregnancies, multiple pregnancies can lead to many difficulties affecting the mother, child, and family [18]. In this sense, the safety of both mothers and newborns should not be put aside in the pursuit of “reasonable” pregnancy rates. In fact, trends of advocating for more natural and affordable fertility methods have been highlighted, and the aim of fertility treatment is shifting from focusing on the pregnancy rate to the birth of healthy singletons [19].

Besides, the cost-effectiveness and patient preferences should also be taken into account. A study on the cost-effectiveness of OS agents (Gn, CC, and LE) for IUI in couples with unexplained subfertility showed that over four IUI-OS cycles conducted within one year, both the cumulative LBR and the average costs per four cycles were highest in cycles with Gn (34.5% and €1809, respectively). And in cycles with CC and LE, the cumulative LBRs were 29.4% and 32%, respectively, and the average costs per four cycles were €362 and €434, respectively [20]. However, evidence from randomized controlled trials comparing the cost-effectiveness of IUI with or without OS is limited, especially in patients with only one patent tube. Theoretically, in terms of cost-effectiveness and patient comfort, IUI without OS should be more easily accepted by patients with a spontaneous ovulatory menstrual cycle.

When comparing the pregnancy outcomes of IUI without OS in patients with unilateral tubal occlusion and patients with bilateral patent tubes, the results showed that the CPR and LBR were comparable, but, interestingly, the first trimester miscarriage rate was significantly higher in patients with unilateral tubal occlusion. After adjusting for female age, BMI, and the duration of infertility, a similar result was obtained. We speculate that in patients with unilateral tubal occlusion, there might be some potential risk factors that could affect pregnancy outcomes, such as chronic endometritis (CE) and endometriosis, which have been suggested to be associated with pregnancy loss. A recent study [21] showed that the highest rate of CE was found in patients with unilateral occlusion (25.0%, *P* = 0.018), followed by women with bilateral occlusion (22.5%) and women with bilateral tubal patency (5.2%). Another study [22] also addressed this association. The authors reported that fallopian tube obstruction was independently associated with CE (OR 3.274, *P* = 0.028). A retrospective cross-sectional study [23] demonstrated that fallopian tube endometriosis is more common than previously suspected, and it showed that the incidence of tubal endometriosis was 11–12% macroscopically and 42.5% microscopically after salpingectomy. Nicolaus et al. [24] reported that women with fallopian tube endometriosis showed a higher rate of tubal occlusion on the same side. In the study conducted by Holzer et al. [21], patients with unilateral occlusion also showed the highest rate (75%) of endometriosis, followed by women with bilateral tubal patency (53.4%) and women with bilateral occlusion (40.9%), although no significant difference was found (*P* = 0.080). In the present study, all included patients had two fallopian tubes, and tubal patency was just diagnosed using HSG/TVS RT-3D-HyCoSy. Those patients with a history of previous surgery (except for caesarean section) were excluded. Therefore, it is difficult to rule out the existence of CE and endometriosis in these patients, as both often presents asymptomatically.

In our study, in the binary logistic regression model, BMI was found to be significantly related to clinical pregnancy and live birth. It is widely accepted that elevated BMI can lead to multiple health issues, including problems with reproduction for women, such as infertility, and pregnancy complications. An abundance of research suggests that obesity has a profound detrimental impact on in vitro fertilization (IVF) pregnancy outcomes. A 2019 meta-analysis [25] that included 21 studies evaluating the association of female obesity with the probability of live birth following IVF demonstrated that female obesity negatively and significantly impacts live birth rates following IVF. Raised BMI is independently associated with higher miscarriage rate after IVF treatment [26]. However, research on whether obesity impacts IUI success have been mixed. Aydin et al. [27] showed that BMI was negatively associated with pregnancies in the first OS IUI cycle. Zheng et al. [28] showed that low BMI was associated with a lower per-cycle LBR of IUI. Besides, Whynott et al. [29] concluded that overweight or obesity does not appear to have a negative effect on live birth after IUI, but obesity may be associated with a higher risk of biochemical pregnancy after IUI. Therefore, further studies on the BMI and IUI pregnancy outcomes were warranted.

There are some limitations in our study. First, the retrospective nature and small sample size of our study may have led to potential inherent biases. Besides, as we mentioned above, IUI without OS might be more cost-effective, and more easily accepted by patients with a spontaneous ovulatory menstrual cycle. But of note, for infertile patients with only one patent tube, if ovulation occurs on the contralateral ovary of the patent fallopian tube, the cycle would be cancelled, considering the potential impact on pregnancy rate and patient preference. Within a certain period time, repeated cycle cancellation might lead to an increase in the cost. Therefore, the cycle cancellation rate should be taken into account. Due to the retrospective nature of our study, the cancellation cycle was not recorded, thus the comparisons of cancellation rates and cost-effectiveness between IUI with or without OS in patients with unilateral tubal occlusion were not possible. And, to our knowledge, no such data have been reported thus far. However, we deduce that in this study, the cycle cancellation rate might be similar between IUI with or without OS in patients with unilateral tubal occlusion. We explain it as follows: Firstly, mild OS is recommended in our center, which means 1–2 dominant follicles developed in most OS cycles. In this study, among 66 OS cycles, 29 OS cycles generated 1 follicle (account for 43.9%), and 32 cycles generated 2 follicles (account for 48.5%). In this case, we infer that in patients with unilateral tubal occlusion, the cancellation rate would not be too much lower in IUI with OS cycles than that in IUI without OS cycles. Additionally, strict cancellation criteria also increased the cancellation rate of OS cycle to some extent. Further studies are warranted to confirm our speculation.

In conclusion, our study showed that IUI without OS might be an alternative treatment strategy for couples affected by unilateral tubal occlusion (diagnosed by HSG or TVS RT-3D-HyCoSy) and male infertility. The pregnancy outcomes were comparable between IUI with or without OS cycles in these patients. However, in IUI without OS cycles, although CPR and LBR were comparable, the first trimester miscarriage rate might be higher in patients with unilateral tubal occlusion than in those with bilateral patent tubes. Further studies are warranted to clarify this relationship.

## Electronic supplementary material

Below is the link to the electronic supplementary material.

Additional file 1

## Data Availability

The data sets used and/or analyzed during the current study are available from the corresponding author upon reasonable request.

## Abbreviations

BMI: Body mass index
CC: Clomiphene citrate
CE: Chronic endometritis
CPR: Clinical pregnancy rate
Gn: Gonadotrophin
IUI: Intrauterine insemination
IVF: In vitro fertilization
LBR: Live birth rate
LE: Letrozole
OS: Ovarian stimulation
HyCoSy: Hysterosalpingo-contrast sonography
HSG: Hysterosalpingography
TVS RT-3D-HyCoSy: Transvaginal real-time three-dimensional hysterosalpingo-contrast sonography
