# Supplementary material for: Pregnancy outcomes of intrauterine insemination without ovarian stimulation in couples affected by unilateral tubal occlusion and male infertility
Source: BMC Pregnancy Childbirth. 2023 May 24;23:376. doi: 10.1186/s12884-023-05705-3 (PMC10207788; doi:10.1186/s12884-023-05705-3)
Supplement: Supplementary file 1 — Additional file 1 [file 12884_2023_5705_MOESM1_ESM.docx]

Supplementary Table

# Pregnancy outcomes of three study groups in the first IUI cycle

|  | **Group A** | **Group B** | **Group C** | ***P*-value (A *vs*. B)** | ***P*-value (A *vs*. C)** |
| --- | --- | --- | --- | --- | --- |
| N | 48 | 32 | 178 |  |  |
| Female age (year) | 31.0 ± 4.0 | 29.5 ± 4.6 | 31.0 ± 4.1 | 0.105 | 0.907 |
| Male age (year) | 33.3 ± 6.3 | 32.0 ± 5.6 | 33.1 ± 5.1 | 0.313 | 0.948 |
| Body mass index (BMI) | 21.3 ± 2.9 | 20.8 ± 2.7 | 21.0 ± 2.8 | 0.776 | 0.683 |
| Type of infertility (n (%)) |  |  |  | 0.854 | 0.596 |
| Primary | 26 (54.2) | 18 (56.3) | 104 (58.4) |  |  |
| Secondary | 22 (45.8) | 14 (43.8) | 74 (41.6) |  |  |
| Duration of infertility (year) | 2.2 ± 1.2 | 2.4 ± 1.3 | 2.8 ± 2.0 | 0.657 | 0.067 |
| Basal FSH (mIU/ml) | 6.3 ± 1.5 | 6.6 ± 2.2 | 6.5 ± 2.2 | 0.572 | 0.675 |
| No. of dominant follicles >16mm | 1.0 ± 0.1 | 1.6 ± 0.7 | 1.0 ± 0.1 | 0.000 ^*^ | 0.319 |
| Endometrium thickness (mm) | 10.1 ± 1.8 | 9.9 ± 2.0 | 10.0 ± 1.4 | 0.367 | 0.717 |
| Total progressive motile sperm count (post-wash) | 19.6 ± 11.6 | 22.3 ± 14.1 | 18.6 ± 11.6 | 0.576 | 0.576 |
| Normal morphology (%, post-wash) | 4.8 ± 2.0 | 4.8 ± 1.8 | 4.4 ± 2.1 | 0.637 | 0.153 |
| tubal occlusion site |  |  |  | 0.267 | / |
| proximal tubal occlusion | 32 (66.7) | 25 (78.1) | / |  |  |
| mid-distal or distal tubal occlusion | 16 (33.3) | 7 (21.9) | / |  |  |
| Clinical pregnancy (n (%)) | 3 (6.3) | 4 (12.5) | 20 (11.2) | 0.429 | 0.424 |
| Live birth (n (%)) | 1 (2.1) | 2 (6.3) | 18 (10.1) | 0.561 | 0.084 |
| Spontaneous abortion (n (%)) | 2(66.7) | 0(0) | 1(5.0) | 0.143 | 0.034 ^*^ |

Group A: IUI without ovarian stimulation (OS) in patients with unilateral tubal occlusion; Group B: IUI with OS in patients with unilateral tubal occlusion; and Group C: IUI without OS cycle in patients with bilateral patent tubes. Values are presented as mean ± standard deviation or n (%).

* *P*<0.05 was considered statistically significant.
